# Characteristics of Medical Products Comprising Human Cells, Genes, or Tissues Developed in Japan and the European Union Compared via Public Assessment Reports

**DOI:** 10.3389/fbioe.2020.606606

**Published:** 2020-12-23

**Authors:** Ryosuke Kurauchi, Hiroi Kasai, Tatsuya Ito

**Affiliations:** ^1^Department of Human Health Science, School of Medicine, Kyoto University, Kyoto, Japan; ^2^School of Medicine, Hokkaido University, Sapporo, Japan; ^3^Institute for Advancement of Clinical and Translational Science, Kyoto University Hospital, Kyoto, Japan

**Keywords:** advanced therapy medicinal products (ATMP), cell therapy, gene therapy, marketing access, regenerative medicine, regenerative medicine products, public assessment reports

## Abstract

Medical products comprising human cells, genes, and tissues have been developed for clinical applications worldwide, and their developmental environment has been established. These products can be imported and exported, but marketing authorization regulations are complicated among regions. This investigation was conducted to identify the characteristics of medical products comprising human cells, genes, and tissues. We used website data, books from survey companies, and reports from public agencies to conduct two investigations. We used website data to conduct a general information survey of 143 cell-therapy and gene-therapy products sold in 24 countries and public assessment reports to individually survey non-clinical and clinical developments of 18 cell-therapy and gene-therapy products developed in Japan and the European Union (EU). The first survey revealed that the numbers of products used in orthopedic surgery and dermatology have increased since 2000, and the numbers of hematological products have increased since 2011. The second investigation revealed that fewer orphaned products were developed in Japan than in the EU. The most appropriate dose was 1.2 × 10^8^ cells per injection per adult. Clinical trials to determine the most appropriate dose were conducted in the EU but not in Japan. No non-clinical immunogenicity tests for autogenous products were conducted in Japan or the EU. Pharmacokinetics tests were not individually performed for sheet-form products. Both *in vivo* and *in vitro* pharmacological tests were more likely to be conducted in the EU, while only one or the other was conducted in Japan. Furthermore, in Japan, carcinogenicity tests were performed based on non-clinical technical guidance, while in the EU, these tests were determined according to each product's features. Fewer clinical trials were performed, and fewer subjects per product were used in Japan than in the EU. Many aspects of the clinical and non-clinical development of medical products comprising human cells, genes, and tissues differ between Japan and the EU. Analyzing these differences will enable the safe and rapid distribution of these products to clinical sites.

## Introduction

Medical products comprising human cells, genes, and tissues (including products approved as transplants and medicinal devices in the past) have been developed for clinical applications worldwide, and their developmental environment has been established (Kondo et al., 2017). From 2014 to 2017, 45 of 143 product developments (31%) were suspended, and half of those were due to plan reformation (BB-Bridge, Inc, 2017). Although plans are frequently reformed when developing new therapies such as cell-based and gene-based therapies, proper development plans that reduce reformation are important for accelerating medical product development. Hanna et al. and Maciulaitis et al. found that most advanced therapy medicinal products (ATMPs), i.e., medical products comprising human cells, genes, and tissues in the European Union (EU), are developed by non-commercial sponsors and small- and medium-sized enterprises (SMEs) rather than by large pharmaceutical companies (Maciulaitis et al., 2012; Hanna et al., 2016). SMEs and start-up companies with biotechnology methods for engineering tissues and modifying genes strive to make their technologies available for clinical applications and lead novel therapies. Large pharmaceutical companies must reduce the cost of developing ATMPs and regenerative medicine products (RMPs), i.e., medical products comprising human cells, genes, and tissues in Japan, because their product development budgets are limited. Product development may avoid failure risk by using good results from research conducted by SMEs (Maciulaitis et al., 2012; Hanna et al., 2016). The number of imported and exported products is expected to increase, and regulatory bodies have attempted to establish common principles (Petricciani et al., 2017); however, developing methods for product approval differs by region. Each region has an expedited program for promoting clinical development including a priority review process and conditional marketing authorization (Detela and Lodge, 2019). For example, Japan has a conditional, time-limited authorization system and “SAKIGAKE” designation scheme (preferential review). The conditional, time-limited authorization is effective for 5–7 years (Daisaku, 2014). The EU has conditional marketing authorization systems and a Priority Medicines (PRIME) scheme. In the EU, conditional marketing authorization is effective for 1 year and is renewed annually (European Medicines Agency, 2020d). Thus, developmental standards for medical products comprising human cells, genes, and tissues remain to be clearly established worldwide. To date, no report has focused on developmental schemes and comparisons of products between Japan and the EU. This study was conducted to review the regulatory and developmental trends for medical products comprising human cells, genes, and tissues in the EU and Japan over two decades. We identified the characteristics of the clinical and non-clinical development of medical products comprising human cells, genes, and tissues between the EU and Japan.

## Materials and Methods

The data sources included information from reports online of survey companies and public agencies. In this study, we conducted two investigations. The first compared the differences in regulations and systems related to medical products comprising human cells, genes, and tissues between the EU and Japan, and we then investigated the number of developed products and their review terms. We used market survey data from BB-Bridge, Inc (2017) and Seed Planning, Inc (2017), and from online information regarding medical products comprising human cells, genes, and tissues (Mitsubishi Chemical Research Corporation, 2009, 2012; Patent Office, 2009; Office of Regulation and System Reform, 2012; Seed Planning, Inc, 2013; Alimchandani, 2017; Arthur D Little, Inc, 2017; Chen et al., 2017; Foundation for Intellectual Property, 2017; Cuende et al., 2018; Shahryari et al., 2019) as of 1 May 2020. We identified 142 cell-based and gene-based therapy products, including products that overlapped or were withdrawn, approved in 24 countries according to disease and approval period. We also surveyed the examination periods of 9 RMPs of the Pharmaceuticals and Medical Devices Agency (PMDA) in Japan (Pharmaceuticals and Medical Devices Agency, 2007; Pharmaceuticals and Medical Devices Agency, 2012a, 2015, 2016a,b, 2019a,b,c,d, 2020a,b; Japan Tissue Engineering Co. Ltd, 2012, 2016, 2019; Terumo Corporation, 2015; JCR Pharmaceuticals Co. Ltd, 2016; AnGes Inc, 2019; Nipro corporation, 2019; Novartis Pharma, 2019) and 19 ATMPs of the European Medicines Agency (EMA) in Europe (European Medicines Agency, 2009, 2011, 2012, 2013, 2014, 2016a,b,c,d, 2017a,b,c, 2018a,b, 2019a,b, 2020a,b), for a total of 28 products, including withdrawn products, as of 1 May 2020. We accessed data sources from public assessment reports of 17 out of 28 products on the EMA or PMDA websites as of 1 May 2020. We also focused on the examination period at each stage of the EMA products using public assessment reports.

In the second investigation, we compared individual product development between Japan and the EU using online product review reports. We examined nonclinical tests and clinical trials for 9 RMPs of the PMDA (Pharmaceuticals and Medical Devices Agency, 2007; Pharmaceuticals and Medical Devices Agency, 2012a, 2015, 2016a,b, 2019a,b,c,d, 2020a,b; Japan Tissue Engineering Co. Ltd, 2012, 2016, 2019; Terumo Corporation, 2015; JCR Pharmaceuticals Co. Ltd, 2016; AnGes Inc, 2019; Nipro corporation, 2019; Novartis Pharma, 2019) and 9 ATMPs of the EMA (European Medicines Agency, 2016a,b,c, 2017a,b,c, 2018a,b, 2019a). Table 1 shows the generic names, brand names, approved countries or regions, developers, and target diseases, as well as whether the drugs were designated as orphan medical products, whether their genes were changed, and whether they were autologous or allogenic as of 1 May 2020. Table 2 provides an overview of the products.

**Table 1:** Medical products comprising human cells, genes, and tissues in 2009–2019 as of 1 May 2020.

**Region**	**Generic name**	**Brand name**	**Disease**	**Company that received the marketing authorization**	**Orphaned drug**	**Gene-transferred**	**Autologous/ allogeneic**
Japan	Human (allogeneic) bone marrow-derived mesenchymal stem cells	Temcell®	Acute graft vs. host disease (GVHD)	JCR Pharmaceuticals Co., Ltd.	○	×	Allogeneic
	Human (autologous) skeletal myoblast-derived cell sheet	HeartSheet®	Severe heart failure	Terumo Corporation	×	×	Autologous
	Human autologous tissue for transplantation	JACC®	Cartilagek defects	Japan Tissue Engineering Co., Ltd.	×	×	Autologous
	Human (autologous) epidermal cell sheet	JACE®	Severe burn Giant congenital melanocytic nevus (GCMN) Dystrophic epidermolysis bullosa	Japan Tissue Engineering Co., Ltd.	Δ^a^	×	Autologous
	Human (autologous) bone marrow-derived mesenchymal stem cells	Stemirac®	Neurological symptoms and dysfunctions by spinal cord injury	Nipro corporation	×	×	Autologous
	Beperminogene perplasmid	Collategene®	Ulcer by chronic arterial occlusion	AnGes, Inc.	×	○	Plasmid
	Human (autologous) corneal limbus-derived mesenchymal stem cells	Nepic®	Limbal stem cell deficiency	Japan Tissue Engineering Co., Ltd.	○	×	Autologous
	Onasemnogene^b^ abeparvovec-xioi	Zolgensma®	Spinal muscular atrophy	Novartis Pharma K.K.	○	○	Adenoaassociated virus
Japan and Europe	Tisagenlecleucel	Kymriah®	Diffuse large B cell lymphoma B cell acute lymphoblastic leukemia	Novartis Pharma K.K. Novartis Europharm Limited	○	○	Autologous
Europe	Allogeneic T cells genetically modified with a retroviral vector encoding for a truncated form of the human low affinity nerve growth factor receptor (ΔLNGFR) and the herpes simplex I virus thymidine kinase (HSV-TK)	Zalmoxis®	GVHD	MolMed S.p.A.	○	○	Allogeneic
	Darvadstrocel	Alofisel®	Anal fistula with Crohn's disease	TiGenix, S.A.U.	○	×	Allogeneic
	Autologous CD34+ enriched cell fraction that contains CD34+ cells transduced with retroviral vector that encodes for the human ADA cDNA sequence	Strimvelis®	Adenosine deaminase (ADA)-severe combined immunodeficiency	GlaxoSmithKline Trading Services	○	○	Autologous
	*Ex vivo*-expanded autologous human corneal epithelial cells containing stem cells	Holoclar®	Limbal stem cell deficiency	Chiesi Farmaceutici S.p.A.	○	×	Autologous
	Spheroids of human autologous matrix-associated chondrocytes	Spherox®	Cartilage defects	Co.don AG	×	×	Autologous
	Axicabtagene ciloleucel	Yescarta®	Diffuse large B cell lymphoma Primary mediastinal B cell lymphoma	Kite Pharma EU B.V.	○	○	Autologous
	Talimogene laherparepvec	Imlygic®	Melanoma	Amgen Europe B.V.	○	○	Herpes simplex virus type 1 (HSV)
	Betibeglogene autotemcel	Zynteglo®	beta-Thalassemia	Bluebird bio (Netherlands) B.V.	○	○	Autologous

a *Not for severe burns, but for GCMN and dystrophic epidermolysis bullosa*.

b *Onasemnogene abeparvovec-xioi was approved by the EMA on 18 May 2020*.

**Table 2:** Overview of medical products comprising human cells, genes, and tissues.

**Country/region**	**Products**	**Product overview**
Japan	Human (allogeneic) bone marrow-derived mesenchymal stem cells (Temcell®)	Mesenchymal stem cells from bone marrow are grown *ex vivo* and injected intravenously. They release cytokines, which suppress T cells. The origin of this product is the same as that of remestemcel-L (Prochymal®).
	Human (autologous) skeletal myoblast-derived cell sheet (HeartSheet®)	Thigh muscle cells are grown *ex vivo* and form a sheet. The product is transplanted on the patient's heart to release cytokines and prevent the heart function from worsening.
	Human autologous tissue for transplantation (JACC®)	Cartilage cells derived from patients arthroscopically are grown *ex vivo* and returned to the articular space to refill the cartilage.
	Human (autologous) epidermal cell sheet (JACE®)	Skin from patients is grown *ex vivo* and transplanted to burned areas lacking skin, treatment for GCMN, or dystrophic epidermolysis bullosa.
	Human (autologous) bone marrow-derived mesenchymal stem cells (Stemirac®)	Mesenchymal stem cells from bone marrow are grown *ex vivo* and injected intravenously. They migrate to the part of damaged neuron, release neurotrophic factors, cause immunoregulation and differentiate into neurons.
	Beperminogene perplasmid (Collategene®)	Plasmids coding human hepatocyte growth factor are injected intramuscularly with an ultrasound guide. Hepatocyte growth factor promotes epithelial cells, particularly vascular endothelial cells, to regenerate and cause angiogenesis.
	Human (autologous) corneal limbus-derived mesenchymal stem cells (Nepic®)	Corneal epithelial stem cells from patients' corneas are grown *ex vivo* and become a sheet, which is transplanted to replenish the cornea. The origin of this product is the same as that of *ex vivo* expanded autologous human corneal epithelial cells containing stem cells (Holoclar®).
	Onasemnogene abeparvovec-xioi (Zolgensma®)	Survival motor neuron gene coding adeno-associated virus 9 is transferred to patients' neurons and muscles to compensate for lacking survival motor neuron proteins.
Japan and EU	Tisagenlecleucel (Kymriah®)	T cells from patients are transferred to the chimeric antigen receptor (CAR) gene and injected intravenously. CAR genes code anti-CD19 that binds to CD19, a B cell lymphoma antigen, 4-1BB (CD137) and CD3 zeta costimulatory domains. When CD19 and anti-CD19 bind, CD3 zeta costimulatory domains activate T cells, grow, obtain effector functions and release inflammatory cytokines and chemokines. 4-1BB promotes CAR T cell growth to remove CD19-positive cells.
EU	Allogeneic T cells genetically modified with a retroviral vector encoding for a ΔLNGFR and the HSV-TK Mut2 (Zalmoxis®)	HSV-derived thymidine kinase is transferred into T cells to be transplanted into patients at high-risk for GVHD. If GVHD develops, the patients take acyclovir or ganciclovir to remove the causative T cells.
	Darvadstrocel (Alofisel®)	Mesenchymal stem cells from adipose tissue are grown *ex vivo* and surgically injected into the anal fistula. They are activated by inflammatory cytokines and prevent lymphocytes from growing and releasing inflammatory cytokines.
	Autologous CD34+-enriched cell fraction that contains CD34+ cells transduced with retroviral vector that encodes for the human ADA cDNA sequence (Strimvelis®)	CD34-positive cells from ADA-severe combined immunodeficiency patients transferred with the ADA gene with virus *ex vivo* are transplanted to refill ADA.
	*Ex vivo* expanded autologous human corneal epithelial cells containing stem cells (Holoclar®)	Patients' corneal epithelial stem cells are grown *ex vivo* and become a sheet, which is transplanted to replenish the corneal cells.
	Spheroids of human autologous matrix-associated chondrocytes (Spherox®)	Cartilage cells are removed arthroscopically and grown *ex vivo*, then returned to the articular space to refill the cartilage.
	Axicabtagene ciloleucel (Yescarta®)	T cells from patients are transferred with the CAR gene and injected intravenously. CAR genes code anti-CD19, CD28, and CD3 zeta costimulatory domains. When CD19 and anti-CD19 bind, CD28 and CD3 zeta costimulatory domains promote T cells to activate, grow, become effector cells and release inflammatory cytokines and chemokines, which remove CD19-positive cells.
	Talimogene laherparepvec (Imlygic®)	The HSV-1 gene is injected locally and transferred into tumor cells to grow and produce human granulocyte macrophage colony-stimulating factor. The drug breaks up tumor cells, releases tumor antigens, and activates systemic antitumor and effector T cell responses.
	Betibeglogene autotemcel (Zynteglo®)	CD34-positive cells from beta-thalassemia patients transferred with the beta-globin gene with lentiviral vector *ex vivo* are transplanted to refill beta-globin.

## Results

### Regulatory Frameworks of Medical Products Comprising Human Cells, Genes, and Tissues in the EU and Japan

Three regulatory bodies regulate the quality, safety, and efficacy of medical products comprising human cells, genes, and tissues using a risk-based approach. Clinical development must be performed under consistent, common rules (e.g., Good Gene, Cellular, and Tissue-based Products Manufacturing Practice, Good Manufacturing Practice, Good Tissue Practice, Good Laboratory Practice, and Good Clinical Practice). However, new marketing authorization schemes and systems for advanced treatments, i.e., cell-based and gene-based therapy products and unmet-need medicines, have recently been introduced in each region. Table 3 summarizes the regulatory frameworks for the two regions.

**Table 3:** Regulatory frameworks in the EU and Japan.

	**EU**	**Japan**
Regulation	Regulation 1394/2007	Pharmaceutical and medical device act
Regulatory body	European Medicines Agency (EMA) and national medicine agencies	Pharmaceuticals and Medical Devices Agency (PMDA)
Official name of medical products comprising human cells, genes or tissues	Advanced Therapy Medical Products (ATMP) in regulation (EC) No 1394/2007	Regenerative Medicine Products (RMPs) in pharmaceutical and medical device act (amended in 2014)
Manufacturing process	Good tissue practice (directive 2004/23/EC), good manufacturing practice (directive 2003/94/EC)	Good manufacturing practice (ordinance 179, 2004), good gene, cellular, and tissue-based products manufacturing practice (ordinance 93, 2014)
Non-clinical development process	Good laboratory practice (directive 2004/10/EC)	Good laboratory practice (ordinance 21, 1997)
Clinical development process	Good clinical practice (directive 2001/20/EC)	Good clinical practice (ordinance 28, 1997)
Priority designation, priority review process, or authorization system	Conditional marketing authorization (commission regulation (EC) No 507/2006) The PRIME (priority medicines) scheme	The SAKIGAKE review system (notification 0401-6 of the PFSB, 2015) Conditional, time-limited authorization (pharmaceutical and medical device act)

In the EU, ATMPs are new treatments for humans based on gene therapy, somatic-cell therapy, or tissue engineering, and the EMA offers conditional marketing authorization and the PRIME scheme (Table 4) (European Medicines Agency, 2020c,d). In Japan, RMPs are defined as human or animal cells processed and used for restructuring, repairing, or forming body structures or functions or treating or preventing diseases and gene-transferred human cells for gene therapy (Pharmaceutical and Medical Device Act, 2016). Since 2014, two schemes have promoted RMP development: the conditional time-limited authorization system and the SAKIGAKE Designation System (Table 4) (Pharmaceutical and Medical Device Act, 2016; Ministry of Health Labour and Welfare, 2019).

**Table 4:** Expedited programs in the EU and Japan.

**Region**	**Program**	
EU	Conditional marketing authorization	Give marketing authorization valid for 1 year if it meets the criteria, such as the risk-benefit balance of products, unmet medical needs for serious diseases, and public health benefits. Requests for renewal must be accompanied by an interim report on how the company has dealt with commitments to carry out required additional research.
	PRIME	Enhance support to develop medicines and reach the market for unmet medical needs. No product was authorized using the PRIME scheme as of 2019.
Japan	Conditional time-limited authorization system	RMPs can be swiftly granted conditional approval for a limited time once their efficacy is predicted and their safety is ensured. RMP criteria include serious diseases, high efficacy, hard-to-conduct confirmatory trials, and expectation of efficacy and safety without confirmatory trials.
	SAKIGAKE designation system	Facilitate the development of innovative drugs, medical devices, and RMPs, leading to their early practical application as the first worldwide. Criteria are products identified as breakthroughs or innovative, serious or life-threatening conditions, high efficacy, and first in class.

### Development and Approval of Medical Products Comprising Human Cells, Genes, and Tissues

**Figure 1:**
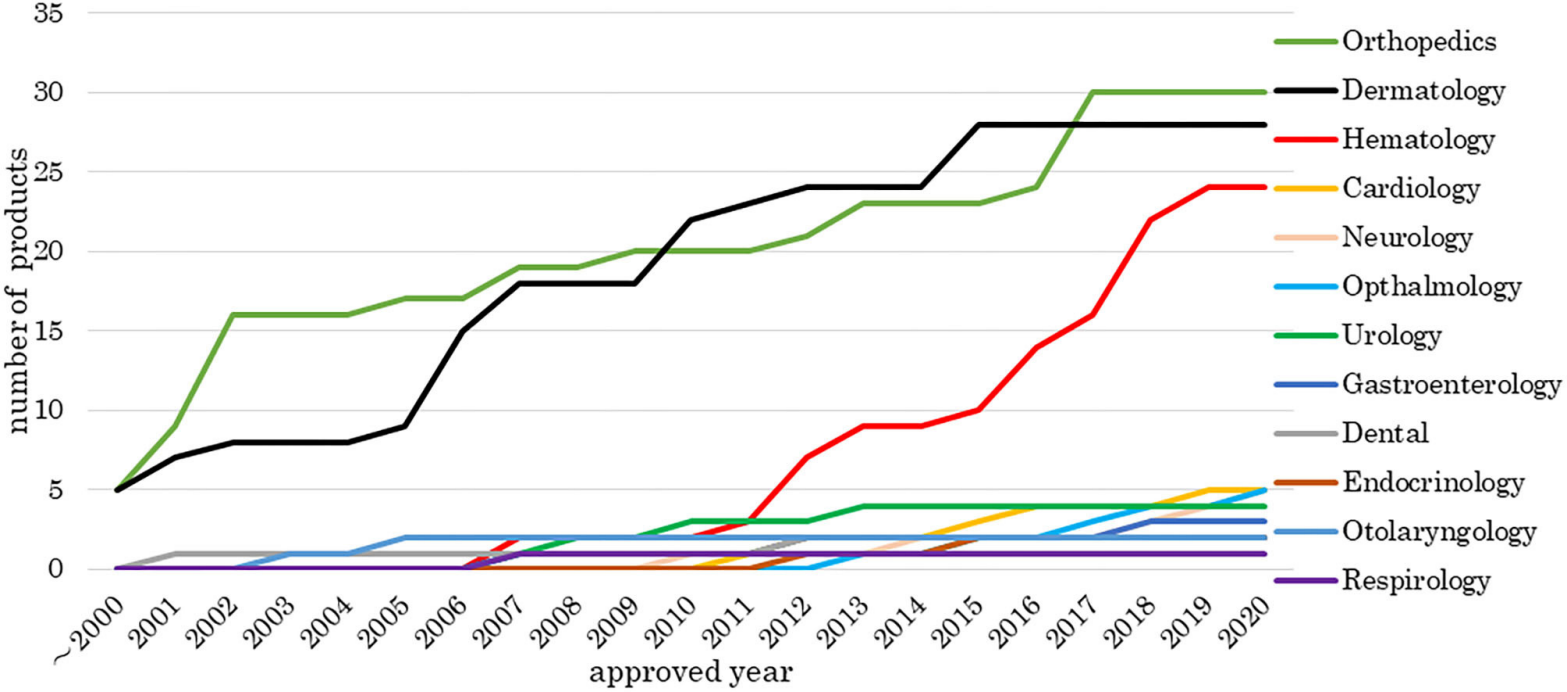
Number of medical products comprising human cells, genes, and tissues by medical field. The data sources included information from online reports of survey companies and public agencies as of 1 May 2020. There were 142 cell-based and gene-based therapy products, including products that overlapped or were withdrawn, approved in 24 countries. The number of products approved in the fields of orthopedic surgery and dermatology has increased since 2000 and the number of hematology-associated products has increased since 2011.

Figure 1 indicates the number of approved medical products comprising human cells, genes, and tissues each year from 2000 to 2020. The number of products approved in the fields of orthopedic surgery and dermatology has increased since 2000, and the number of hematology-associated products has increased since 2011. Other medical fields currently have fewer numbers of approved products.

**Figure 2:**
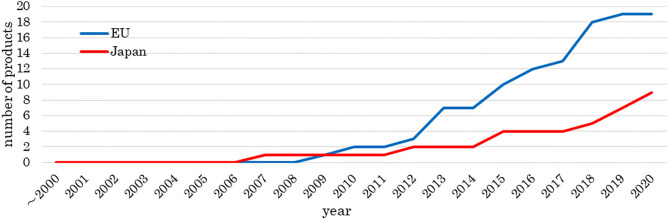
Number of approved medical products comprising human cells, genes, and tissues. The data sources included information from online reports of survey companies and public agencies as of 1 May 2020. The number of approved products is increasing.

**Figure 3:**
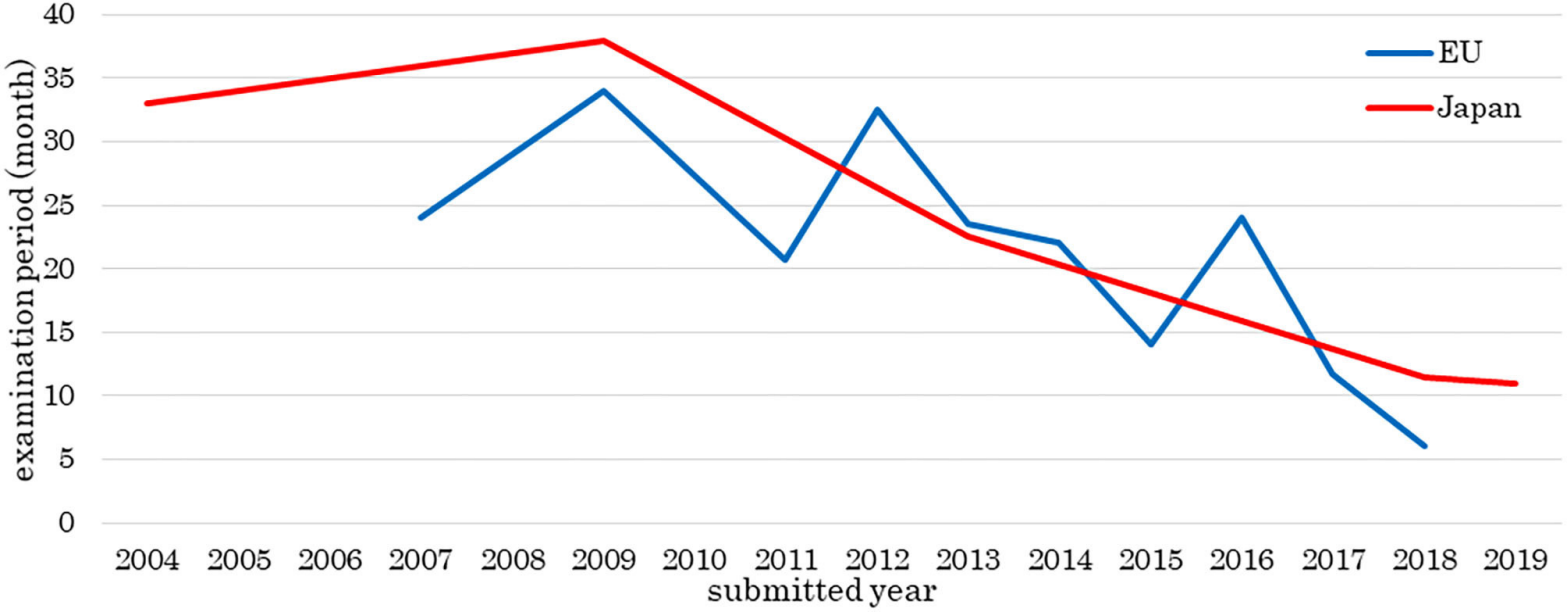
Examination period of medical products comprising human cells, genes, and tissues. The data sources included information from the assessment reports of public agencies as of 1 May 2020. The review periods tended to become shorter over the two decades. The horizontal line shows the year of a products submission, not approval.

Figure 2 shows the number of approved medical products comprising human cells, genes, or tissues, and Figure 3 shows the review times for the EU and Japan over two decades. Although the number of approved products differs between the EU and Japan, their review periods tended to shorten over two decades.

The review times in 2009, 2012, and 2016 were longer than those of other years. Alipogene tiparvovec (Glybera®) was assessed three times, and more time was taken to answer the list of questions or the list of outstanding issues for spheroids of human autologous matrix-associated chondrocytes (Spherox®) or darvadstrocel (Alofisel®) than other products.

### General Information on RMPs and ATMPs

We focused on approved RMPs and ATMPs in Japan and the EU. The PMDA and EMA designated five of nine products (56%) and eight of nine products (89%) as orphan medical products, respectively. The SAKIGAKE Designation System reviewed human (autologous) bone marrow-derived mesenchymal stem cells (Stemirac®), and the SAKIGAKE Designation System and PRIME scheme reviewed onasemnogene abeparvovec-xioi (Zolgensma®) (January 2017–July 2019). In Japan, three of nine products (33%) were gene transferred; in the EU, six of nine (67%) were gene transferred. In Japan, 6 of 9 products (67%) were autogenic; in the EU, six of nine (67%) were autogenic.

Table 5 summarizes the products' injection doses and shows the number of cells injected by body weight. The most suitable liquid dose was 1.2 × 10^8^ cells per injection per adult, indicating that the most appropriate RMP and ATMP doses were independently decided according to disease type. In Japan, clinical trials for determining the best dose were conducted only for onasemnogene abeparvovec-xioi (Zolgensma®) and tisagenlecleucel (Kymriah®). In the EU, these trials were not conducted for the three ATMPs: an autologous CD34^+^ enriched cell fraction that contains CD34^+^ cells transduced with a retroviral vector encoding the human adenosine deaminase (ADA) complementary deoxyribonucleic acid (cDNA) sequence (Strimvelis®), *ex vivo* expanded autologous human corneal epithelial cells containing stem cells (Holoclar®) and betibeglogene autotemcel (Zynteglo®). In Japan, the dose was determined from non-clinical data; in the EU, the dose was determined from clinical data.

**Table 5:** Doses of medical products comprising human cells, genes, and tissues.

**Country/region**	**Products**	**Cells injected per body weight (cells per kg)**	**Cell numbers for 60-kg adults (cells)**	**Product characteristics**	**Data for dose selection**
Japan	Human (allogeneic) bone marrow-derived mesenchymal stem cells (Temcell®)	2 × 10^6^	1.2 × 10^8^	8–12 injections	Nonclinical trials Overseas equivalent product
	Human (autologous) skeletal myoblast-derived cell sheet (HeartSheet®)	–	3.75 × 10^8^	Independent of weight/sheet form	Non-clinical trials
	Human autologous tissue for transplantation (JACC®)	Not referred	–	–	Not referred
	Human (autologous) epidermal cell sheet (JACE®)	Not referred	–	Sheet form	Calculated from body surface area per surgery
	Human (autologous) bone marrow-derived mesenchymal stem cells (Stemirac®)	3.34 × 10^6^	0.5–2 × 10^8^	–	Non-clinical trials Bibliographic survey (for pediatric)
	Beperminogene perplasmid (Collategene®)	–	0.5 mg/parts	Gene therapy 2–3 injections	Non-clinical trials 2 doses, phase II trial and clinical study
	Human (autologous) corneal limbus-derived mesenchymal stem cells (Nepic®)	–	–	Sheet form	Not referred
	Onasemnogene abeparvovec-xioi (Zolgensma®)	1.1 × 10^14^ vector genome/kg	–	Gene therapy	2 and 3 doses, phase I trials
Japan and EU	Tisagenlecleucel (Kymriah®) (for B cell acute lymphoblastic leukemia)	0.2–5.0 × 10^6^	1.2–2.5 × 10^8^	2.5 × 10^8^ (independent of weight over 50 kg)	Phase I/II trials Phase II clinical trials for chronic lymphocytic leukemia (dose titration method)
	Tisagenlecleucel (Kymriah®) (for diffuse large B cell lymphoma)	–	0.6–6 × 10^8^	Independent of weight	Clinical trials (dose titration method)
EU	Allogeneic T cells genetically modified with a retroviral vector encoding for a ΔLNGFR and the HSV-TK Mut2 (Zalmoxis®)	1 × 10^7^	6 × 10^8^	1–3 injections	2 doses, phase I/II trials
	Darvadstrocel (Alofisel®)	2 × 10^6^	1.2 × 10^8^	–	2 doses, phase I/II clinical trials
	Autologous CD34+ enriched cell fraction that contains CD34+ cells transduced with retroviral vector that encodes for the human ADA cDNA sequence (Strimvelis®)	2–20 × 10^6^	1.2–12 × 10^8^	For pediatrics	Pediatric hematopoietic stem cell transplantation (bibliographic survey)
	*Ex vivo*-expanded autologous human corneal epithelial cells containing stem cells (Holoclar®)	79,000–31,600/cm^2^	–	Sheet form	Not referred
	Spheroids of human autologous matrix-associated chondrocytes (Spherox®)	10–70/cm^2^	–	Spheroid injection	3 doses, phase II trials
	Axicabtagene ciloleucel (Yescarta®)	1.0–2.0 × 10^6^	0.6–1.2 × 10^8^	Permitted multiple injections	Phase I clinical trials (dose titration method)
	Talimogene laherparepvec (Imlygic®)	10^6^ PFU/ml or 10^8^ PFU/ml	–	Oncolytic virus Permitted multiple injections	3 doses, phase I clinical trials
	Betibeglogene autotemcel (Zynteglo®)	5–20 × 10^6^	3–12 × 10^8^	–	The minimum recommended dose was based on practice for autologous transplantation, and the maximum was not decided.

### Comparison of Non-clinical Trials Between Japan and EU

Tables 6, 7 summarize the results of non-clinical trials and carcinogenicity tests, respectively. Neither Japan nor the EU tested the immunogenicity of autogenous products, but they both conducted allogeneic testing. For talimogene laherparepvec (Imlygic®), a genetically modified herpes simplex virus (HSV) for tumor lysis, beperminogene perplasmid (Collategene®), a plasmid-coding hepatocyte growth factor, and onasemnogene abeparvovec-xioi (Zolgensma®), a genetically modified adeno-associated virus 9, no studies have investigated whether host cells immunologically recognize these drugs as foreign substances. Thirteen of 18 products (72%) underwent non-clinical trials with animal disease models. Regarding non-clinical pharmacology tests, no *in vivo* tests were conducted in Japan if no appropriate animal model was available. Four of nine products (57%) in Japan and eight of nine products (88%) in the EU underwent non-clinical *in vitro* trials for efficacy. Thus, both *in vivo* and *in vitro* tests were likely to be conducted in the EU, whereas only one or the other was likely to be conducted in Japan. Pharmacokinetics tests were not individually conducted for sheet-form products, except for human (autologous) skeletal myoblast-derived cell sheets (Heartsheet®) and human (autologous) corneal limbus-derived mesenchymal stem cells (Nepic®), but these tests were individually conducted for all liquid-form products except human autologous tissue for transplantation (JACC®) and autologous CD34^+^ enriched cell fractions that contain CD34^+^ cells transduced with a retroviral vector encoding the human ADA cDNA sequence (Strimvelis®). For nonclinical carcinogenicity tests, six of nine products were investigated by karyotyping, soft-agar colony testing, and *in vivo* tumor-formation testing following technical guidance in Japan (Pharmaceuticals and Medical Devices Agency, 2016c). In the EU, karyotyping and soft-agar colony tests were performed on only two products, *in vivo* tumor-formation testing was performed on six products, and carcinogenicity was tested using various methods such as testing for gene expression involving carcinogenicity. Thus, in Japan, some tests were performed based on non-clinical technical guidance, whereas in the EU, trial contents were considered according to purpose, administration form, and the location of each administered product.

**Table 6:** Implementation status of non-clinical trials.

**Country/ region**	**Products**	**Autologous/ allogeneic**	**Immunogenicity tests**	**Use of disease model animals**	**Non-clinical *in vitro* trials for efficacy**	**Non-clinical *in vivo* trials for efficacy**	**Sheet product**	**Non-clinical trial for pharmacokinetics**
Japan	Human (allogeneic) bone marrow-derived mesenchymal stem cells (Temcell®)	Allogeneic	○	×	○	Undone because there were no model animals	×	○
	Human (autologous) skeletal myoblast-derived cell sheet (HeartSheet®)	Autologous	Not referred	○	Not referred	○	○	Non-clinical trials for pharmacology and toxicology
	Human autologous tissue for transplantation (JACC®)	Autologous	Not referred	○	Not referred	○	×	Not referred
	Human (autologous) epidermal cell sheet (JACE®)	Autologous	Not referred	×	Bibliographic research	Bibliographic research	○	Not referred
	Human (autologous) bone marrow-derived mesenchymal stem cells (Stemirac®)	Autologous	Not referred	○	○	○	×	○
	Beperminogene perplasmid (Collategene®)	Plasmid	Antigenicity test performed	○	○	○	×	○
	Human (autologous) corneal limbus-derived mesenchymal stem cells (Nepic®)	Autologous	Not referred	○	Not referred	○	○	Non-clinical trials for pharmacology and toxicology
	Onasemnogene abeparvovec-xioi (Zolgensma®)	Virus	Not referred	○	Not referred	○	×	○
Japan and EU	Tisagenlecleucel (Kymriah®)	Autologous	Not referred	○	○	○	×	○
EU	Allogeneic T cells genetically modified with a retroviral vector encoding for a ΔLNGFR and the HSV-TK Mut2 (Zalmoxis®)	Allogeneic	○	○	○	○	×	○
	Darvadstrocel (Alofisel®)	Allogeneic	○	×	○	○	×	○
	Autologous CD34+ enriched cell fraction that contains CD34+ cells transduced with retroviral vector that encodes for the human ADA cDNA sequence (Strimvelis®)	Autologous	Undone for autogenic product	×	○	○	×	Investigated by injecting umbilical cord blood
	*Ex vivo*-expanded autologous human corneal epithelial cells containing stem cells (Holoclar®)	Autologous	Not referred	×	Bibliographic research	Bibliographic research	○	Bibliographic research
	Spheroids of human autologous matrix-associated chondrocytes (Spherox®)	Autologous	Undone for autogenic and local use product	○	○	○	×	○
	Axicabtagene ciloleucel (Yescarta®)	Autologous	Not referred	○	○	○	×	○
	Talimogene laherparepvec (Imlygic®)	Virus	Not referred	○	○	○	×	○
	Betibeglogene autotemcel (Zynteglo®)	Autologous	Not referred	○	○	○	×	○

**Table 7:** Implementation status of carcinogenicity in non-clinical trials.

**Country/region**	**Products**	**Karyotyping**	**Soft-agar colony test**	**Tumor formation *in vivo***	**Expressed gene analysis**	**Others**
Japan	Human (allogeneic) bone marrow-derived mesenchymal stem cells (Temcell®)	○	○	○	×	–
	Human (autologous) skeletal myoblast-derived cell sheet (HeartSheet®)	○	○	○	×	–
	Human autologous tissue for transplantation (JACC®)	○	○	○	×	–
	Human (autologous) epidermal cell sheet (JACE®)	○	○	○	×	–
	Human (autologous) bone marrow-derived mesenchymal stem cells (Stemirac®)	○	○	○	×	–
	Beperminogene perplasmid (Collategene®)	×	×	○	×	Effect on tumor cell growth and metastasis
	Human (autologous) corneal limbus-derived mesenchymal stem cells (Nepic®)	○	○	○	×	–
Japan and EU	Tisagenlecleucel (Kymriah®)	×	×	○	×	Lack of gene-induced cell growth *in vitro*
EU	Onasemnogene abeparvovec-xioi (Zolgensma®)	×	×	○	×	–
	Allogeneic T cells genetically modified with a retroviral vector encoding for a ΔLNGFR and the HSV-TK Mut2 (Zalmoxis®)	×	×	○	○	Cell survival ratio without growth factors Number of injected cells Investigation of viral introduction pattern
	Darvadstrocel (Alofisel®)	○	○	○	○	–
	Autologous CD34+ enriched cell fraction that contains CD34+ cells transduced with retroviral vector that encodes for the human ADA cDNA sequence (Strimvelis®)	×	×	×	×	–
	*Ex vivo*-expanded autologous human corneal epithelial cells containing stem cells (Holoclar®)	○	○	×	×	–
	Spheroids of human autologous matrix-associated chondrocytes (Spherox®)	×	×	×	○	Infiltration or not
	Axicabtagene ciloleucel (Yescarta®)	×	×	○	○	–
	Talimogene laherparepvec (Imlygic®)	×	×	×	×	Reports of HSV carcinogenicity
	Betibeglogene autotemcel (Zynteglo®)	×	×	○	○	Integration site analysis

### Clinical Trials for RMPs and ATMPs in Japan and EU

Table 8 lists subject numbers and clinical trials. The median numbers of subjects in Japan and the EU were 32 and 182, respectively. The median numbers of clinical trials in Japan and the EU were three and five, respectively. Conditional, time-limited authorization or conditional marketing authorization enables applicants to obtain marketing authorization without confirmatory trials, as with human (autologous) skeletal myoblast-derived cell sheets (Heartsheet®), human (autologous) bone marrow-derived mesenchymal stem cells (Stemirac®), beperminogene perplasmid (Collategene®), allogeneic T cells genetically modified with a retroviral vector encoding a truncated form of the human low affinity nerve growth factor receptor (ΔLNGFR), herpes simplex I virus thymidine kinase (HSV-TK Mut2) (Zalmoxis®), *ex vivo* expanded autologous human corneal epithelial cells containing stem cells (Holoclar®), and betibeglogene autotemcel (Zynteglo®).

**Table 8:** Features of clinical trials.

**Country/region**	**Products**	**Amount of subjects**	**Amount of clinical trials and studies*^b^***
Japan	Human (allogeneic) bone marrow-derived mesenchymal stem cells (Temcell®)	39^a^	3^a^
	Human (autologous) skeletal myoblast-derived cell sheet (HeartSheet®)	26	3
	Human autologous tissue for transplantation (JACC®)	32	2
	Human (autologous) epidermal cell sheet (JACE®)	2 (severe burn)	1
		8 (GCMN)	2
		9 (epidermolysis bullosa)	3
	Human (autologous) bone marrow-derived mesenchymal stem cells (Stemirac®)	17	2
	Beperminogene perplasmid (Collategene®)	241	10
	Human (autologous) corneal limbus-derived mesenchymal stem cells (Nepic®)	22	2
	Onasemnogene abeparvovec-xioi (Zolgensma®)	70	5
Japan and EU	Tisagenlecleucel (Kymriah®)	265	5
EU	Allogeneic T cells genetically modified with a retroviral vector encoding for a ΔLNGFR and the HSV-TK Mut2 (Zalmoxis®)	86	2
	Darvadstrocel (Alofisel®)	236	2
	Autologous CD34+ enriched cell fraction that contains CD34+ cells transduced with retroviral vector that encodes for the human ADA cDNA sequence (Strimvelis®)	37	6
	*Ex vivo*-expanded autologous human corneal epithelial cells containing stem cells (Holoclar®)	182	5
	Spheroids of human autologous matrix-associated chondrocytes (Spherox®)	625	18
	Axicabtagene ciloleucel (Yescarta®)	181	5
	Talimogene laherparepvec (Imlygic®)	588	8
	Betibeglogene autotemcel (Zynteglo®)	104	6

a *386 subjects and 6 trials counting clinical trials of equivalent products*.

b *Includes bibliographic investigations*.

**Figure 4:**
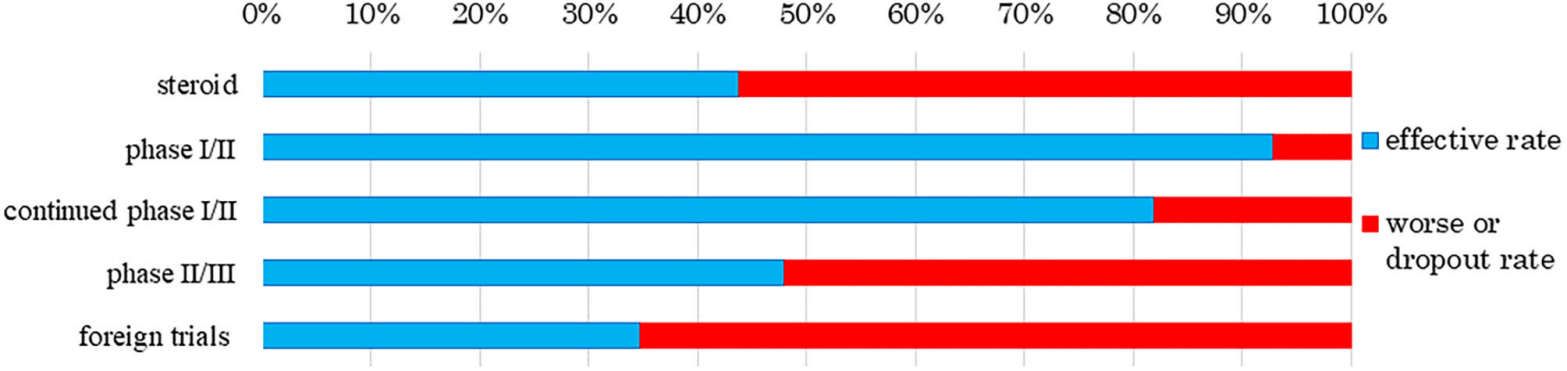
Response rate to human (allogeneic) bone marrow-derived mesenchymal stem cells (Temcell®). The data sources were assessment reports and application materials for human (allogeneic) bone marrow-derived mesenchymal stem cells (Temcell®), which are used for corticosteroid-refractory acute GVHD following hematopoietic stem cell transplantation. The foreign trials were performed using an equivalent product, remestemcel-L (Prochymal®) in the USA, Canada, Australia, Germany, Spain, and the UK. The effective rate decreased as the trial phase progressed. The rate of steroid use was 43.8% as reported by Murata et al. (2013). While 13 subjects showed effective treatment in a phase I/II trial of human (allogeneic) bone marrow-derived mesenchymal stem cells (Temcell®), one subject worsened or dropped out. In the continued phase I/II trial, 12 subjects showed effective treatment and two worsened or dropped out. Twelve subjects showed effective treatment in the phase II/III trial and 13 worsened or dropped out. In a trial of remestemcel-L (Prochymal®) in foreign countries, 60 subjects showed effective treatment whereas 113 had worsened disease.

**Figure 5:**
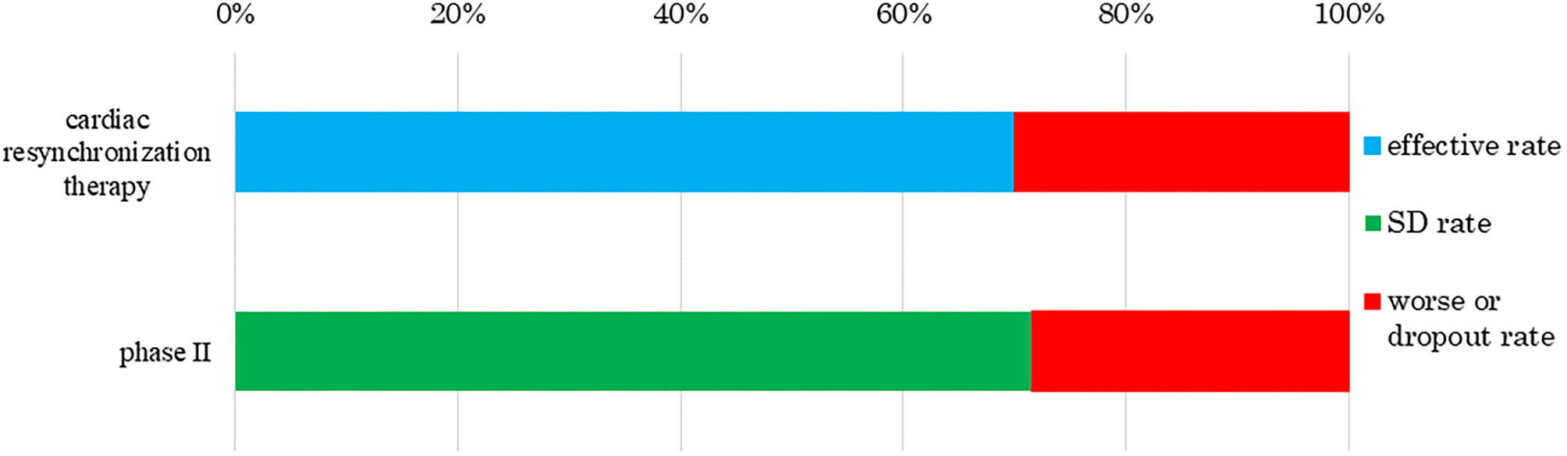
Response rate to human (autologous) skeletal myoblast-derived cell sheets (HeartSheet®). The data sources were assessment reports and application materials for human (autologous) skeletal myoblast-derived cell sheets (Heartsheet®). Cardiac resynchronization therapy was used for patients with severe heart failure who did not respond to standard drug therapies. Human (autologous) skeletal myoblast-derived cell sheets (Heartsheet®) were used for patients with severe heart failure unresponsive to standard treatments including drug and invasive therapies. This treatment mediated its effect by stabilizing cardiovascular function, i.e., stable disease (SD). The rate of cardiac resynchronization therapy was 70%, as reported by Birnie and Tang (2006). No subject showed effective treatment in a phase II trial of human (autologous) skeletal myoblast-derived cell sheets (Heartsheet®), but five achieved SD and two worsened or dropped out.

We also analyzed clinical trial results for the rates of effectiveness, stable disease (SD), ineffectiveness, and number of dropout subjects for human (allogeneic) bone marrow-derived mesenchymal stem cells (Temcell®) and human (autologous) skeletal myoblast-derived cell sheets (Heartsheet®) with conventional treatments including steroid (Murata et al., 2013) and cardiac resynchronization therapy (Birnie and Tang, 2006) (Figures 4, 5). Interestingly, in the clinical trials for human (allogeneic) bone marrow-derived mesenchymal stem cells (Temcell®), the effective rate decreased as the trial phase progressed (Figure 4), which is unusual in medical product development. In the phase II/III trial in Japan, the applicant did not set a control group, but an appropriated control group was used in other countries. After the phase II/III trial, the applicant found that the severity of the inclusion criteria in these clinical trials differed between Japan and the USA. Thus, they compared the active drug group in Japan with the reset control group, whose degree of patient severity was consistent between Japan and the USA. Therefore, the effective rate in the phase II/III clinical trial was higher than that in the reset control group.

Human (autologous) skeletal myoblast-derived cell sheets (Heartsheet®) were assessed by their ability to stabilize cardiovascular function (Figure 5). The trial subjects had severe heart failure, which can only be effectively treated by heart transplantation. A PMDA officer wrote, as a personal opinion, “through the assessment, human (autologous) skeletal myoblast-derived cell sheets (Heartsheet®) have a certain effect on typical drug-resistant severe heart failure (European Medicines Agency, 2008; Maruyama, 2015).” Therefore, human (autologous) skeletal myoblast-derived cell sheets (Heartsheet®) secured conditional time-limited approval, although it did not improve patients' heart function. As with human (autologous) skeletal myoblast-derived cell sheets (Heartsheet®), clinical issues must be clarified, and effectiveness standards must be defined when developing medical products comprising human cells, genes, and tissues.

## Discussion

We investigated the development and approval of medical products comprising human cells, genes, and tissues worldwide. Numbers of medical products comprising human cells, genes, and tissues in orthopedics and dermatology were increased at the beginning of the 2000's and in hematology in 2011. Most orthopedic products aim to refill cartilage, which tends to be damaged through traumatic or sports-related injury. Injected medical products comprising human cells, genes, and tissues remain in the joint cavity, which has no blood vessels. These products are unlikely to spread into the systemic circulation even if undifferentiated cells are present; therefore, the risk of tumorigenesis is low. Dermatological products are locally administered as sheets, which can easily and comparatively cure patients, for example, by reducing inflammation. Thus, orthopedic and dermatological products were the primary products being developed. Seven hematological products were approved from 2011 to 2013, of which five were for hematopoietic stem cell transplantation. At that time, it was simple to obtain approval, and this made it easier to develop medical products comprising human cells, genes, and tissues. Since then, the number of products in hematology has increased. Furthermore, the numbers of medical products comprising human cells, genes, and tissues approved by the EU and Japan have increased. Among the two agencies, the review times have decreased, while the median approval time for new active substances has not significantly changed (Centre for Innovation in Regulatory Science, 2019), indicating these trends are similar between the two agencies. In the EMA, more time was taken in 2009, 2012, and 2016 than in other years. “Steps taken for the assessment of the product” stated in the public assessment report shows which review stages took longer. Alipogene tiparvovec (Glybera®), the first gene therapy product in the EU, was submitted by UniQure Biopharma B.V. in 2009. Alipogene tiparvovec (Glybera®) was assessed three times. The first assessment started on 23 December 2009, and the Committee for Human Medicinal Products issued a negative opinion on 23 June 2011. It was then reexamined from 23 June to 20 October 2011. The third assessment started on 23 January 2012, and the Committee for Human Medicinal Products finally issued a positive opinion on 19 July 2012 (European Medicines Agency, 2012). Thus, the review term was 2.5 years. For spheroids of human autologous matrix-associated chondrocytes (Spherox®), submitted by Co.don AG in 2012, the list of questions took longer to answer than those of other products in 2013 (European Medicines Agency, 2017b) because the biopharmaceutical company had to collect clinical trial data. It also took longer to answer the list of outstanding issues for darvadstrocel (Alofisel®), submitted by Tigenix, S.A.U. in 2016, than for other products in 2016 (European Medicines Agency, 2017a). The public assessment report showed that the EMA found no difference in the quality of life between the darvadstrocel (Alofisel®) and control groups and that all patients in the clinical trials received the same dose regardless of the number of fistulas.

Clinical trials to determine suitable cell doses are seldom conducted in Japan but are required in the EU. The PMDA requires an investigation of the most appropriate dose and usage, which seems to be effective throughout drug development in terms of efficacy, while developers must pay attention to dose-dependent risks and the risk of immune responses caused by repeat injections (Pharmaceuticals and Medical Devices Agency, 2016c). According to the EMA guidelines, different doses should be applied, and the dose-response relationship should be tested if different doses cannot be applied (European Medicines Agency, 2008, 2018c). The EMA is likely to require a stricter investigation of a dose than the PMDA. Furthermore, Japan and the EU might consider non-clinical immunogenicity tests for autogenous products unnecessary. Guidance in Japan suggests that although non-clinical immunogenicity tests are required for allogenic and autogenic products, they are not always conducted for self-recognizing autogenic products (Pharmaceuticals and Medical Devices Agency, 2000; Pharmaceuticals and Medical Devices Agency, 2008a,b,c,d, 2012b,c). EMA guidelines do not describe autogenic product immunogenicity tests. This allows developers to save costs related to immunogenicity tests. As a result, autogenic products are cheaper than allogenic products (Shukla et al., 2019). In the guidance and guidelines, conventional stand-alone tests for pharmacokinetics are unnecessary in Japan and the EU (Pharmaceuticals and Medical Devices Agency, 2016c; European Medicines Agency, 2017d). However, when products are used directly or target specific organs, developers in Japan must clarify the locality and the impact on efficacy and safety through pharmacokinetic tests (Pharmaceuticals and Medical Devices Agency, 2000; Pharmaceuticals and Medical Devices Agency, 2008a,b,c,d, 2012b,c), and in the EU, biodistribution data may not be required if the cellular distribution potential is thought to be limited (European Medicines Agency, 2018c). Thus, in Japan, but not the EU, biodistribution data for sheet-form products must always be investigated.

Only *in vitro* or *in vivo* non-clinical pharmacological tests are conducted in Japan, while both are conducted in the EU. In Japan, applicants can submit the results of either *in vitro* or *in vivo* tests to suggest product mechanisms. A proof of concept must be shown by using an experimental animal model or suitable cells as per the PMDA guidance (Pharmaceuticals and Medical Devices Agency, 2008a,b,c,d, 2012b,c). Similarly, if relevant animal models cannot be developed, *in vitro* studies may replace animal studies as per the EMA guidelines (European Medicines Agency, 2017d). To date, no products have been approved without non-clinical *in vivo* tests in the EU except for *ex vivo* expanded autologous human corneal epithelial cells containing stem cells (Holoclar®). Regarding non-clinical pharmacological tests, the EMA seems to undertake a stricter review process compared with the PMDA.

Other regulations are related to carcinogenicity. The PMDA provides simple integrated guidance, which is easily understood for conducting testing (Pharmaceuticals and Medical Devices Agency, 2016c), whereas the EU guidelines state that tests must be performed along with written product characteristics (European Medicines Agency, 2008, 2018c). Clinical trials in Japan used fewer subjects than those in the EU. The number of subjects can be compensated for in two ways: using the results of clinical studies from other countries and postmarketing clinical trials for conditional, time-limiting approval or conditional marketing authorization. The former was the case for human (allogeneic) bone marrow-derived mesenchymal stem cells (Temcell®), which are equivalent to remestemcel-L (Prochymal®), whose clinical trials were conducted in the USA for rare diseases. Using many subjects from other countries improved the limited results obtained using fewer subjects in Japan. Postmarketing clinical trials for conditional, time-limiting approval were imposed on human (autologous) skeletal myoblast-derived cell sheets (Heartsheet®), beperminogene perplasmid (Collategene®), and human (autologous) bone marrow-derived mesenchymal stem cells (Stemirac®), which were only developed in Japan. Their safety was assessed via the results of a phase II trial, which secured a conditional, time-limiting approval. The confirmatory efficacy was investigated in a postmarketing clinical trial. In the EU, *ex vivo* expanded autologous human corneal epithelial cells containing stem cells (Holoclar®), allogeneic T cells genetically modified with a retroviral vector encoding ΔLNGFR and HSV-TK Mut2 (Zalmoxis®) and betibeglogene autotemcel (Zynteglo®) received conditional marketing authorization because *ex vivo* expanded autologous human corneal epithelial cells containing stem cells (Holoclar®) were approved with retrospective data, and allogeneic T cells genetically modified with a retroviral vector encoding ΔLNGFR and HSV-TK Mut2 (Zalmoxis®) and betibeglogene autotemcel (Zynteglo®) were approved with limited clinical data (Detela and Lodge, 2019). Furthermore, it is difficult to define the effectiveness of medical products comprising human cells, genes, and tissues in clinical trials, i.e., when maintaining heart function in the human (autologous) skeletal myoblast-derived cell sheet (Heartsheet®) clinical trials and when determining similar criteria between domestic and overseas trials for human (allogeneic) bone marrow-derived mesenchymal stem cells (Temcell®). Tisagenlecleucel (Kymriah®) was developed in Japan and the EU. The nonclinical tests and clinical data reviewed by the two regulatory bodies were similar although there are some different points in the guidelines between Japan and the EU. This utilization of the non-clinical and clinical data can save developmental costs. However, the review term by the PMDA in Japan was longer than the target review period in the SAKIGAKE scheme, which caused a delay in approval in Japan. If the developers apply EU data to a review by the PMDA, they should recognize the differences between guidelines and consult with the PMDA from an early stage of product development (Pharmaceuticals and Medical Devices Agency, 2019a).

Overall, EMA guidelines undergo stricter investigation than those of the PMDA and many products have been developed in compliance with these guidelines; however, some products in the EU and Japan have been developed without conducting their required tests. The developers must consult with regulatory agencies and judged to have effectively conducted the relevant examinations. The developers also utilize the SAKIGAKE scheme or PRIME scheme for fast track applications. Especially, the PRIME scheme is a supportive system for SMEs and academia. Products designated for the PRIME scheme can apply for accelerated assessment (European Medicines Agency, 2020c) but are automatically designated for the SAKIGAKE scheme (Ministry of Health Labour and Welfare, 2019). This difference may affect the development schedule of medical products comprising human cells, genes, and tissues. In addition, the review term of the SAKIGAKE scheme was shorter than that of the PRIME scheme (median time = 5.1 and 8 months, respectively) (Office of Pharmaceutical Industry Research, 2020). These points are the disadvantages of the PRIME scheme compared with the SAKIGAKE scheme. Furthermore, the SAKIGAKE scheme has other advantages including accompaniment consultation and the potential for conditional approval. In terms of conditional approval, developers in the EU must renew it annually, whereas conditional and time-limited approval in Japan is effective for 5–7 years. In addition, national insurance reimbursement is paid for products even under conditional approval in Japan. Therefore, developers can use their funds for further development in Japan compared with the EU. However, a disadvantage of the SAKIGAKE scheme is that it is designated only for products that are developed first in Japan. This makes it difficult for companies outside Japan, which do not have a Japanese subsidiary, to apply through the SAKIGAKE scheme (Nagai, 2019).

We focused on the characteristics of medical products comprising human cells, genes, and tissues developed over two decades. Our study had some limitations. First, the number of products was small. The numbers of cell-therapy medicinal products and *in vivo* gene therapy products were only 15 and 3, respectively. To increase accuracy, we will investigate products approved by the Food and Drug Administration and the Therapeutic Goods Administration (the Australian regulatory agency) and products that were once approved by the EMA but have since been withdrawn. Second, we did not study products developed worldwide, just those in the EU and Japan. The first gene therapy product was developed in the USA followed by China, Russia, and the Philippines (Seed Planning, Inc, 2017; Shahryari et al., 2019). To date, many products have been developed in the USA and South Korea. Third, we conducted this research with limited information obtained from development data and review results online and from documents. Based on this research, we suggest that the development pattern of medical products comprising human cells, genes, and tissues is extremely complex for each product, and researchers are perhaps rushing to summarize the findings from their experience and knowledge; however, a more measured approach might be required to establish their development patterns.

## Conclusion

EMA guidelines require stricter investigations than those of the PMDA. Although there are similar fast track systems in Japan and the EU, the details of each system, for example, the designation conditions, are different. The results of this study combined with further investigations will enable the rapid and safe clinical application of medical products comprising human cells, genes, and tissues.

## Data Availability Statement

The original contributions presented in the study are included in the article/supplementary materials, further inquiries can be directed to the corresponding author/s.

## Author Contributions

RK conceived this research, collected and analyzed the data for the EU and Japan, and drafted the manuscript. HK and TI analyzed and coordinated this research. All authors read and approved the final manuscript.

## Conflict of Interest

The authors declare that the research was conducted in the absence of any commercial or financial relationships that could be construed as a potential conflict of interest.
